# 12-year analysis of incidence, microbiological profiles and in vitro antimicrobial susceptibility of infectious keratitis: the Nottingham Infectious Keratitis Study

**DOI:** 10.1136/bjophthalmol-2020-316128

**Published:** 2020-06-24

**Authors:** Darren Shu Jeng Ting, Charlotte Shan Ho, Jessica Cairns, Ahmad Elsahn, Mouhamed Al-Aqaba, Tim Boswell, Dalia G Said, Harminder Singh Dua

**Affiliations:** 1 Academic Ophthalmology, University of Nottingham, Nottingham, UK; 2 School of Medicine, University of Nottingham, Nottingham, UK; 3 Department of Microbiology, Nottingham University Hospitals NHS Trust, Nottingham, UK; 4 Ophthalmology, Nottingham University Hospitals NHS Trust, Nottingham, UK; 5 Division of Ophthalmology and Visual Sciences, University of Nottingham, Nottingham, UK

**Keywords:** cornea, epidemiology, infection, microbiology, ocular surface

## Abstract

**Background/aims:**

To examine the incidence, causative microorganisms and in vitro antimicrobial susceptibility and resistance profiles of infectious keratitis (IK) in Nottingham, UK.

**Methods:**

A retrospective study of all patients who were diagnosed with IK and underwent corneal scraping between July 2007 and October 2019 (a 12-year period) at a UK tertiary referral centre. Relevant data, including demographic factors, microbiological profiles and in vitro antibiotic susceptibility of IK, were analysed.

**Results:**

The estimated incidence of IK was 34.7 per 100 000 people/year. Of the 1333 corneal scrapes, 502 (37.7%) were culture-positive and 572 causative microorganisms were identified. Sixty (4.5%) cases were of polymicrobial origin (caused by ≥2 different microorganisms). Gram-positive bacteria (308, 53.8%) were most commonly isolated, followed by Gram-negative bacteria (223, 39.0%), acanthamoeba (24, 4.2%) and fungi (17, 3.0%). *Pseudomonas aeruginosa* (135, 23.6%) was the single most common organism isolated. There was a significant increase in *Moraxella* spp (p<0.001) and significant decrease in *Klebsiella* spp (p=0.004) over time. The in vitro susceptibilities of Gram-positive and Gram-negative bacteria to cephalosporin, fluoroquinolone and aminoglycoside were 100.0% and 81.3%, 91.9% and 98.1%, and 95.2% and 98.3%, respectively. An increase in resistance against penicillin was observed in Gram-positive (from 3.5% to 12.7%; p=0.005) and Gram-negative bacteria (from 52.6% to 65.4%; p=0.22).

**Conclusion:**

IK represents a relatively common and persistent burden in the UK and the reported incidence is likely underestimated. Current broad-spectrum antimicrobial treatment provides a good coverage for IK, although challenged by some level of antimicrobial resistance and polymicrobial infection.

## Introduction

Infectious keratitis (IK) represents a major cause of corneal blindness globally, accounting for over 5% of all blindness.^1^ It has also been estimated to cause 1.5–2.0 million monocular blindness each year.^1^ It is a common yet potentially sight-threatening ophthalmic emergency, characterised by corneal ulceration, epithelial defect and/or stromal infiltrate. Based on the limited evidence in the literature, the incidence of IK has been estimated at 0.04–8.0 per 1000 people per year, with a substantially higher rate noted in developing countries such as India, Nepal and Burma.^1^

A wide array of microorganisms, including bacteria, fungi, viruses and parasites, notably acanthamoeba, have been implicated in IK. In view of the diverse causative microorganisms and potentially rapid clinical progression, intensive broad-spectrum antimicrobial treatment, either with cephalosporin/aminoglycoside dual therapy or fluoroquinolone monotherapy, is usually commenced to provide an initial comprehensive coverage for IK.^2 3^ Uncommonly, adjuvant therapies such as tetracyclines (protease inhibitors), amniotic membrane transplantation and the recently introduced modality of therapeutic photoactivated chromophore for keratitis-corneal cross-linking may be required to halt the progression of IK.^4–7^

The diagnosis of IK is primarily made on clinical grounds, supplemented by microbiological investigations such as corneal scraping for microscopy, culture and sensitivity testing.^1^ Depending on the geographical and temporal variations, the profile of causative microorganisms of IK may differ significantly across different regions.^8^ For instance, fungi were shown to be the most common organism for IK in China and India whereas bacteria were most commonly identified in the USA and the UK.^1^ In addition, the in vitro antimicrobial susceptibility and resistance of ocular isolates similarly varied significantly across the world, with the rate of methicillin-resistant *Staphylococcus aureus* (MRSA) ranging from 0.1% to 36.6%.^9 10^ Moreover, the proportion of multidrug resistant (MDR) ocular isolates is reportedly rising in some regions.^10^

To date, there are only two studies in the literature that reported the incidence of IK in the UK, which was estimated at 3.6–52.1 per 100 000 population/year during the period of 1995–2006.^11 12^ A number of studies have recently examined the microbiological profiles and/or in vitro antibiotic susceptibility and resistance profiles of IK in the UK.^9 13–15^ Within the region of Nottingham, UK, the most recent review on IK was conducted during the period of 2007–2010 and only focussed on severe and sight-threatening cases.^16^

In this study, we aimed to provide an up-to-date and comprehensive analysis on the incidence, microbiological profiles and in vitro antimicrobial susceptibility and resistance of IK in Nottingham, UK, over the past 12 years and to compare the findings with the recent literature.

## Materials and methods

This was a retrospective study of all patients who were diagnosed with IK and underwent corneal scraping between July 2007 and October 2019 (a 12-year period) at the Queen’s Medical Centre (QMC), Nottingham, UK. Cases were identified through the local microbiology electronic database. QMC was the only tertiary referral centre for managing ophthalmic diseases in Nottingham. The eye casualty embedded within the QMC was open 24/7 to manage patients with emergency ophthalmic conditions, including IK. There were two other nearby hospitals in the East Midlands regions, including Derby Royal Hospital and Kings Mill Hospital, but they covered a different subset of the population and were not included in Nottingham population or our IK database.

Based on the departmental guideline for IK, all patients presented with moderate-sized corneal ulcers (>1 mm diameter) or atypical presentation of corneal ulcer were subjected to microbiological investigation, which included corneal scraping for microscopy (with Gram staining), microbiological culture and sensitivity testing. Corneal scrapes were inoculated on chocolate agar (for fastidious organisms), blood agar (for bacteria) and Sabouraud dextrose agar (for fungi). For suspected cases of acanthamoeba keratitis, non-nutrient *Escherichia coli*-enriched agar plate was used for inoculation. All cultures were incubated for at least 1 week (and up to 3 weeks for suspected acanthamoeba keratitis). The identity of the microorganisms was confirmed through standard culture and bacteriology tests. For example, *S. aureus* was identified by cultural characteristics and positive Pasteurex test whereas *Streptococcus pneumonia* was identified by cultural characteristics and sensitivity to optochin disc. Corneal scraping was repeated in the same eye when the patient was unresponsive to treatment regardless of positive or negative outcome of the first culture. These cases were only counted as one clinical episode.

Causative microorganisms were categorised into Gram-positive and Gram-negative bacteria, fungi and acanthamoeba. Polymicrobial keratitis was defined as IK caused by two or more types of microorganisms simultaneously during the same infective episode. Combined cefuroxime and gentamicin/amikacin were used for deemed sight-threatening keratitis (greater than 1 mm lesion, location within the central 6 mm zone and/or related to contact lens wear); or levofloxacin monotherapy for non-sight-threatening keratitis (infiltrate size of 1 mm or less, peripheral location and not related to contact lens wear) was the first-line antimicrobial therapy used during the entire study period. In vitro antimicrobial susceptibility and resistance were determined using the standard disc diffusion assay or Microscan (Beckman Coulter, Indianapolis, United States) and interpreted according to the clinical breakpoints set by the European Committee on Antimicrobial Susceptibility Testing.^17^ MDR was defined as resistance to three or more classes of antibiotic.

The population in Nottingham was estimated at between 300 000 and 328 000 people during the study period (https://www.ukpopulation.org/nottingham-population/), and these figures were used to estimate the incidence of IK within the region of Nottingham, UK. For study years of 2007 and 2019 (without the full-year data), the incidence was extrapolated from 6 months’ and 10 months’ data, respectively. This was because the electronic database was only introduced in July 2007 and the study was concluded in October 2019.

Ethical approval was waived by the local research ethics committee as this retrospective study was classified as a service evaluation (reference number: 19-265C). The study was conducted in accordance with the tenets of Declaration of Helsinki.

### Statistical analysis

For descriptive and analytic purposes, the study was divided into two time periods, 2007–2013 (which included the study period of previous study)^16^ and 2014–2019. Statistical analysis was performed using SPSS V.26.0 (IBM SPSS Statistics for Windows). Comparison between groups was conducted using Pearson’s Chi-square or Fisher’s exact test where appropriate for categorical variables and unpaired t-test or Mann-Whitney U test for continuous variables. Normality of data distribution was assumed if the skewness and kurtosis z-values were between −1.96 and +1.96 and the Shapiro-Wilk test p value was >0.05. All continuous data were presented as mean±SD and/or 95% CI. Pearson’s correlation coefficient (r) analysis was performed to examine the incidence of IK over time and was interpreted as follows: weak (r=0.00–0.40), moderate (r=0.41–0.69) and strong (r=0.70–1.00), with negative values being interpreted in the same way.^18^ P value of <0.05 was considered statistically significant.

## Results

### Overall description and incidence of IK

**Figure 1:**
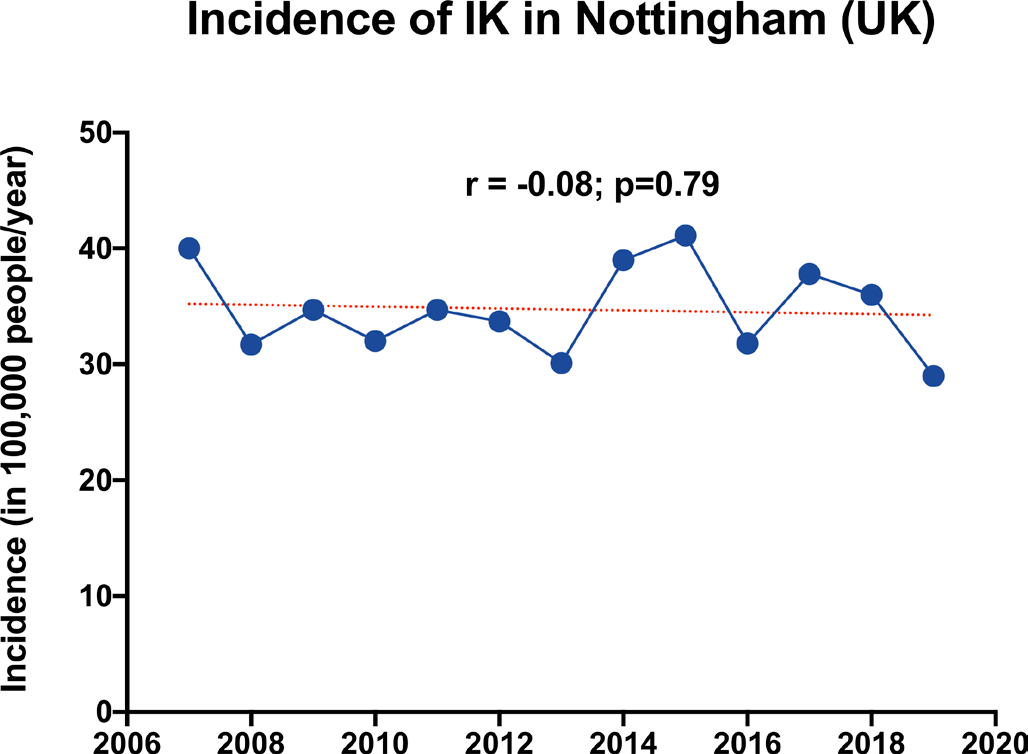
The pattern of incidence of infectious keratitis in the region of Nottingham, UK, between 2007 and 2019. IK, infectious keratitis.

During the 12-year study period, 1400 corneal scrapes were performed in patients with IK; the mean age was 49.9±22.2 years and 50.4% were men. There were 67 cases where repeat corneal scrapings were performed in the same eye. On no occasion were both cultures positive. After excluding 67 repeat corneal scrapings, there were a total of 1333 cases of IK. The overall incidence of IK in our region was estimated at 34.7 per 100 000 population/year (95% CI, 32.4 to 37.1 per 100 000 population/year), with a stable trend observed over time (r=−0.08; p=0.79; figure 1).

### Types of causative organisms

Of all 1333 cases, 502 (37.7%) were culture-positive and 572 causative microorganisms were identified (table 1). Gram-positive bacteria (308, 53.8%) were most commonly isolated, followed by Gram-negative bacteria (223, 39.0%), acanthamoeba (24, 4.2%) and fungi (17, 3.0%). In terms of specific isolates, *Pseudomonas aeruginosa* (135, 23.6%), *S. aureus* (91, 15.9%) and *Streptococci* spp (77, 13.5%) were the three most common causative microorganisms identified. Sixty (4.5%) cases were of polymicrobial origin (caused by ≥2 different microorganisms), with 50 (3.8%) cases having two causative microorganisms and 10 (0.8%) cases having three causative microorganisms. Of the 60 cases, the majority (57, 95%) were mixed bacteria/bacteria infection with only 3 (5%) cases of mixed fungi/bacteria infection. The most common combination of isolates for polymicrobial cases was *Streptococci* spp combined with coagulase-negative staphylococcus (9, 15%). There was a significant increase in *Moraxella* spp (from 2.8% to 10.0%; p<0.001) and significant decrease in *Klebsiella* spp (from 3.5% to 0.3%; p=0.004) over time.

**Table 1:** Summary of microbiological profiles of infectious keratitis in Nottingham, UK, between 2007–2013 and 2014–2019

Organisms	2007–2019	2007–2013	2014–2019	P value*
	n=572; n (%)	n=282; n (%)	n=290; n (%)	
Gram-positive	308 (53.8)	153 (54.3)	155 (53.4)	0.53
*S. aureus*	91 (15.9)	49 (17.4)	42 (14.5)	0.34
CoNS	75 (13.1)	39 (13.8)	36 (12.4)	0.64
*Streptococci* spp	77 (13.5)	37 (13.1)	40 (13.8)	0.74
Bacilli	63 (11.0)	28 (9.9)	35 (12.1)	0.35
Others†	2 (0.3)	0 (0.0)	2 (0.7)	0.5
Gram-negative	223 (39.0)	108 (38.3)	115 (39.7)	0.74
*P. aeruginosa*	135 (23.6)	67 (23.8)	68 (23.4)	0.66
*Moraxella* spp	37 (6.5)	8 (2.8)	29 (10.0)	<0.001
*Klebsiella* spp	11 (1.9)	10 (3.5)	1 (0.3)	0.004
Others‡	40 (7.0)	23 (8.2)	17 (5.9)	0.21
Fungi	17 (3.0)	10 (3.5)	7 (2.4)	0.43
Yeast	10 (1.7)	6 (2.1)	4 (1.4)	0.91
Filamentous	7 (1.2)	4 (1.4)	3 (1.0)	0.91
Acanthamoeba	24 (4.2)	11 (3.9)	13 (4.5)	0.72

*Chi-square or Fisher’s exact test (if any variable was <5) was used to detect any significant changing trend of the microbiological profiles between 2017–2013 and 2014–2019. The analysis was performed at two levels; the first level evaluated the changes among Gram-positive and Gram-negative organisms, fungi and acanthamoeba; and the second level examined the changes of the subtypes of the organisms within the four groups. Significant p values (<0.05) are underlined.

†Others include *Enterococci* spp.

‡Others Include *Achromobacter* spp, *Acinetobacter* spp, *Citrobacter koseri*, *Enterobacter* spp, *Kingella* spp, *Serratia marcescens*, *Haaemophilus* spp, *Proteus* spp, *Neisseria* spp and *Stenotrophomonas maltophilia*.

CoNS, coagulase-negative staphylococcus.

### In vitro antimicrobial susceptibility and resistance profiles

The in vitro antimicrobial susceptibilities for cephalosporin, fluoroquinolone and aminoglycoside were 100.0% (25/25), 91.9% (205/223) and 95.2% (177/186) for Gram-positive bacteria; and 81.3% (65/80), 98.1% (212/216) and 98.3% (174/177) for Gram-negative bacteria (table 2). From 2007–2013 to 2014–2019, there was an increase in resistance against penicillin in Gram-positive (from 3.5% to 12.7%; p=0.005) and Gram-negative bacteria (from 52.6% to 65.4%; p=0.22). There were only four (0.3%) MDR isolates and one (0.07%) MRSA noted in this study. Our first-line treatment, either with combined therapy (cephalosporin and aminoglycoside) or fluoroquinolone monotherapy, provided good antibiotic coverage for 97.3% (n=396/407) and 95.2% (n=418/439) of the cases, respectively.

**Table 2:** Summary of antibiotic susceptibility of infectious keratitis in Nottingham, UK, between 2007–2013 and 2014–2019

Organisms	2007–2019	2007–2013	2014–2019	P value*
	n (%)	n (%)	n (%)	
Gram-positive				
Penicillin†	260/283 (91.9)	136/141 (96.5)	124/142 (87.3)	0.005
Cefuroxime	25/25 (100.0)	17/17 (100.0)	8/8 (100.0)	1.0
Gentamicin	177/186 (95.2)	97/101 (96.0)	80/85 (94.1)	0.73
Ciprofloxacin	164/182 (90.1)	92/100 (92.0)	72/82 (87.8)	0.35
Levofloxacin	41/41 (100.0)	16/16 (100.0)	25/25 (100.0)	1.0
Gram-negative				
Penicillin†	36/80 (45.0)	18/38 (47.4)	18/52 (34.6)	0.22
Cefuroxime	65/80 (81.3)	30/38 (78.9)	35/42 (83.3)	0.62
Amikacin	172/174 (98.9)	91/92 (98.9)	81/82 (98.8)	1.0
Gentamicin	174/177 (98.3)	93/94 (98.9)	81/83 (97.6)	0.6
Ciprofloxacin	175/179 (97.8)	92/94 (97.9)	83/85 (97.6)	1.0
Levofloxacin	53/53 (100.0)	16/16 (100.0)	37/37 (100.0)	1.0

*Chi-square or Fisher’s exact test (if any variable was <5) was performed to determine the significant difference between the two time periods. Significant p value is underlined.

†Penicillin group includes penicillin, amoxicillin and flucloxacillin.

Antibiotic susceptibility of the four most commonly isolated microorganisms of IK, including *P. aeruginosa*, *S. aureus*, *Streptococci* spp and coagulase-negative staphylococcus, is summarised in table 3. All these organisms were generally susceptible (>90%) to the commonly used cephalosporin (ie, cefuroxime), aminoglycosides and fluoroquinolones used in our study.

**Table 3:** Summary of antibiotic susceptibility of the four most common microorganisms of IK in Nottingham, UK, during 2007–2019

Antibiotics	*P. aeruginosa*	*S. aureus*	*Streptococci* spp	CoNS
	n (%)	n (%)	n (%)	n (%)
Penicillin*	0/1 (0.0%)	89/90 (98.9)	68/71 (95.8)	61/73 (83.6)
Cefuroxime	–	17/17 (100.0)	4/4 (100.0)	–
Gentamicin†	133/134 (99.3)	90/90 (100.0)	4/4 (100.0)	68/73 (93.2)
Amikacin†	133/133 (100.0)	2/2 (100.0)	–	–
Ciprofloxacin‡	133/134 (99.3)	82/90 (91.1)	–	66/73 (90.4)
Levofloxacin‡	6/6 (100.0)	–	39/39 (100.0)	–

The percentage shown refers to the antibiotic susceptibility rate of each microorganisms. Rate of resistance is equivalent to 100% minus the antibiotic susceptibility rate.

*Penicillin group includes penicillin, amoxicillin and flucloxacillin.

†Gentamicin and amikacin are aminoglycosides and usually one or the other was tested.

‡Ciprofloxacin and levofloxacin are fluoroquinolone and usually one or the other was tested.

CoNS, coagulase-negative staphylococcus; IK, infectious keratitis.

## Discussion

IK represents a major cause of corneal blindness worldwide, particularly in the developing countries. To the best of our knowledge, this represents the third study in the UK that reported the incidence as well as the causative microorganisms and in vitro antibiotic susceptibility and resistance profiles of IK.

### Incidence

Currently, there is limited literature reporting on the incidence of IK globally. This is mainly due to the fact that most studies reported the incidence/prevalence of corneal blindness without distinguishing the underlying causes such as infective, inflammatory, traumatic, degenerative and others.^1^ In this study, we observed a stable trend of IK in Nottingham, UK, over the past decade (2007–2019), with an estimated incidence of 34.7 per 100 000 population/year. This figure is comparable to the incidence previously reported in Portsmouth, UK, which was 40.1–52.1 per 100 000 population/year during 1997–2006, and substantially higher than the rate reported in the West of Scotland, which was 3.6 per 100 000 population/year during 1995. Consistent with the literature, the incidence of IK observed in our study was considerably lower than the rate in developing countries such as India and Nepal, which was estimated at 1.1–8.0 per 1000 people (or 110–799 per 100 000 population/year).^19 20^ Such significant variation of the incidence is primarily related to the population-based risk factors such as agricultural industry, high-risk occupation (with increased risk to corneal trauma), poorer environmental and personal hygiene, lower level of education and poorer access to sanitation and healthcare in the developing countries.^1^

It is noteworthy to mention that the reported incidence of IK in our study and some other studies are likely to be underestimated as it was based on patients with IK who had undergone corneal scraping.^11^ Corneal scraping is usually performed in patients with moderate/severe IK with sizeable infiltrate where adequate sampling was possible or in patients with mild IK where the clinical presentation was atypical. Based on our local departmental protocol, all patients with a corneal infiltrate of >1 mm or those with atypical infection were subjected to corneal scraping. This means that patients with mild and typical IK were not included in this study. In addition, viral keratitis cases were not captured in this study as the majority of cases were treated based on the typical clinical appearance of dendritic ulcer without any microbiological investigation. Nonetheless, the relatively stable incidence of IK observed in our study during the past decade suggests that IK represents a relatively common and persistent burden in the UK.

### Microbiological profiles

Causative microorganisms of IK are subjected to wide geographical variations across the world.^8^ A systematic review of 36 studies demonstrated that bacteria were the most common isolates in developed countries whereas fungi were most commonly reported in developing countries.^8^ The recent Asia Cornea Society Infectious Keratitis Study, which was conducted in Asia and included over 6000 patients with IK, demonstrated that fungi were the most common group of causative microorganism in China and India whereas bacteria were the most common organism in developed countries such as Singapore.^21^ Another large study conducted in the Southern China similarly reported a predominance of fungal keratitis in the region.^22^ The variation of microorganisms is likely influenced by various factors, including the occupational risk of corneal trauma, agricultural industry, use of contact lens, national income and others.^1 8^

In our study, we observed that Gram-positive bacteria were the most common group of microorganisms responsible for IK during the entire study period. This finding parallels the results of many other studies conducted in the UK (table 4)^9 13–15^ and other countries.^23–25^ Within the UK, several studies^9 14 15^ have observed that coagulase-negative staphylococcus was most commonly isolated, which was in contrast to our study where *Pseudomonas* spp was the main causative organism (table 4). This could be related to the differences in contact lens wear in different population groups, a fact that was not explored in our study and some other studies.^9 13–15^ Interestingly, we observed a significant increase in trend in *Moraxella* keratitis in our region that was similar to other regions in the UK such as Sunderland^9^ and Manchester,^14^ suggesting a potentially emerging endemic issue within the UK. In addition, polymicrobial keratitis presents unique diagnostic and therapeutic challenges to the clinicians as the treatment outcome is often variable and the treatment course is prolonged.^26 27^ We observed 4.5% cases were of polymicrobial keratitis in our study, which was lower than the rate reported in the literature (10%–14%).^9 14 22^ It would be interesting and clinically valuable to examine the clinical outcomes of these polymicrobial cases as evidence on this area remains scarce.^28^

**Table 4:** Summary of the microbiological profiles and antibiotic susceptibility of infectious keratitis in the UK between 2010 and 2019 (based on year of publication)

Year	Authors	Study period	Region	Total CS	Culture positivity (%)	Microbiological profiles*	Antibiotic susceptibility (%)†			
							PEN	CEF	AMG	FQ
2011	Orlans *et al* 13	1999–2009	Oxford	467	54.0	*Pseudomonas* spp (28.5%); CoNS (25.8%); *S. aureus* (12.4%)	50.4 (P)	80.9 (P); 8.7 (N)	87.2 (P); 100 (N)	85.4 (P); 99.0 (N)
2017	Tan *et al* 14	2004–2015	Manchester	4229	32.6	CoNS (24.4%); *S. aureus* (15.1%); *Streptococci* (13.3%)	–	86.6 (P); 61.4 (N);	88.8 (P); 96.5 (N)	83.1 (P); 90.8 (N)
2018	Ting *et al* 9	2008–2017	Sunderland	914	44.5	CoNS (25.9%); *S. aureus* (13.6%); *Streptococci* (12.1%)	–	–	–	–
2019	Tavassoli *et al* 15	2006–2017	Bristol	2614	38.1	CoNS (36.0%); *Pseudomonas* spp (15.8%); *Streptococci* (7.0%)	95.0–100 (P); 31.0 (N)	–	100 (P); 97.0–100 (N)	91.0–100 (P); 97.0–100 (N)
2020	Ting *et al* 9 (current study)	2007–2019	Nottingham	1333	37.7	*Pseudomonas* spp (23.6%); *S. aureus* (15.9%); *Streptococci* 13.5%)	91.9 (P); 45.0 (N)	100 (P); 81.3 (N)	95.2 (P); 98.3–98.9 (N)	90.1–100 (P); 97.8–100 (N)

*The three most common microorganisms isolated in the study.

†P = Gram-positive bacteria; n=Gram-negative bacteria.

AMG, aminoglycosides (include gentamicin and amikacin); CEF, cefuroxime; CoNS, coagulase-negative staphylococci; CS, corneal scrapes; FQ, fluoroquinolones (include ciprofloxacin, ofloxacin and levofloxacin); PEN, penicillin.

Our culture positivity rate was shown to be 37.7%, which was comparable to some studies^14 15 23^ but lower than the others.^9 13^ Plausible explanations for the relatively low culture yield include possible use of antibiotic before the visit to hospital, inadequate sampling from the infected corneas and a lower threshold for performing corneal scrapes in non-infective cases, including sterile corneal melt and marginal keratitis. For patients who were already on any antibiotics before the hospital visit, our standard practice was to stop all the antibiotics for 24–48 hours before performing any corneal scrapes. Therefore, it is likely that any prior use of antibiotics would have lesser impact than expected on the culture yield.

### Antibiotic susceptibility and resistance

Antimicrobial resistance (AMR) is emerging as a global health threat of 21st century. AMR has been increasingly reported in both systemic and ocular infections.^1 29^ In our study, we observed a substantial increase in penicillin resistance in both Gram-positive (12.7%) and Gram-negative bacteria (65.4%). However, most of the bacterial isolates, including the most common organisms, were susceptible to the current broad-spectrum antibiotics (ie, cephalosporin/aminoglycoside dual therapy and fluoroquinolone monotherapy), which was similarly reported in other parts of the UK (table 4).^14 15^ Reassuringly, there were only four (0.3%) MDR isolates and one (0.07%) MRSA identified in our study.

Nonetheless, AMR in relation to IK is emerging as a serious concern in other parts of the world, including China USA^22 25^ and India.^30 31^ For instance, the rate of MRSA ocular isolates was reported to be in the range of 0.1%–5.0% in the UK^9 14 15^ whereas Antibiotic Resistance Monitoring in Ocular micRoorganisms (ARMOR) Study conducted in the USA reported a substantially higher rate (36.6%) of MRSA ocular isolates.^10^ Peng *et al* 25 observed that 35% of the ocular isolates were resistant to moxifloxacin and the rate increased over time. Similarly, Oldenburg *et al* 30 and Lalitha *et al* 31 reported a significant increase in fluoroquinolone (ofloxacin/moxifloxacin) resistance among *S. aureus* and *P. aeruginosa* isolated in South India. In addition, there was a significant increase in the number of MRSA from 2002 to 2013 in the same region.^31^ The discrepancy in the AMR rate in ocular isolates observed among different regions may be related to the difference in the prescribing practice (eg, inappropriate and overuse of chloramphenicol eye drops for non-bacterial eye infection), choice of antibiotics used, environmental transmission and genomic variations in the causative microorganisms. In addition, the variation in the antibiotic susceptibility testing method employed in different studies might have an influence on the reported results; for instance, broth microdilution minimum inhibitory concentration assays were used to determine the antibiotic susceptibility in the ARMOR Study^10^ whereas standard disc diffusion assays and/or Microscan were used in our study and other studies.^13 15^

### Strengths and limitations

This study provides an up-to-date examination on the incidence of IK in one region of the UK. However, the incidence was calculated based on the number of IK cases that had corneal scrapings performed, thereby the incidence was likely underestimated. A prospective study with inclusion of all presumed IK, including those without corneal scraping, could help ascertain the incidence of IK in the future. In addition, the true representation of the causative microorganisms is currently challenged by the low-to-moderate yield of the conventional microbiological investigation such as corneal scraping. Although our culture positivity rate (37.7%) was comparable to some studies, the moderate diagnostic yield highlights the need for further improvement.^1^ This issue can be potentially ameliorated by other emerging investigative techniques such as in vivo confocal microscopy,^32 33^ PCR and/or next generation sequencing,^34 35^ which have demonstrated their values in the diagnosis and clinical decision making in challenging IK cases.

Current broad-spectrum antibiotics provide good treatment coverage in most IK cases; however, not all the antibiotics used were subjected to antibiotic susceptibility testing. In addition, analysis of the susceptibility of chloramphenicol—an over-the-counter antibiotic treatment that is routinely prescribed in primary care—was not possible as this was not routinely examined in our unit. Reassuringly, a recent UK study did not observe any significant increase in resistance against this antibiotic.^14^ As the commonly used topical antibiotics in ophthalmology differs from other specialties, a close collaboration with the microbiology department to standardise the in vitro antimicrobial susceptibility testing for IK would provide a more comprehensive evaluation of the susceptibility and resistance profiles. Although it is beyond the scope of our current study, it would be valuable to examine other clinically relevant aspects such as any prior use of antibiotics, causes (eg, contact lens wear, trauma and so on) and outcomes of IK, in a future study.

In conclusion, IK represents a relatively common and persistent burden in the UK and the reported incidence is likely to be underestimated. Current broad-spectrum antimicrobial treatment provides good coverage for IK, although challenged by some level of AMR and polymicrobial infection. Future surveillance of the incidence, causative microorganisms and antimicrobial susceptibility resistance with well-designed prospective studies would be beneficial.
